# A disease category feature database construction method of brain image based on deep convolutional neural network

**DOI:** 10.1371/journal.pone.0232791

**Published:** 2020-06-01

**Authors:** Yanli Wan, Xifu Wang, Quan Chen, Xingyun Lei, Yan Wang, Chongde Chen, Hongpu Hu

**Affiliations:** 1 Institute of Medical Information, Chinese Academy of Medical Sciences & Peking Union Medical College, Beijing, China; 2 School of Traffic and Transportation, Institute of System Engineering and Control, Beijing Jiaotong University, Beijing, China; Polytechnical Universidad de Madrid, SPAIN

## Abstract

**Background:**

Constructing a medical image feature database according to the category of disease can achieve a quick retrieval of images with similar pathological features. Therefore, this approach has important application values in the fields such as auxiliary diagnosis, teaching, research, and telemedicine.

**Methods:**

Based on the deep convolutional neural network, an image classifier applicable to brain disease was designed to distinguish between the image features of the different brain diseases with similar anatomical structures. Through the extraction and analysis of visual features, the images were labelled with the corresponding semantic features of a specific disease category, which can establish an association between the visual features of brain images and the semantic features of the category of disease which will permit to construct a disease category feature database of brain images.

**Results:**

Based on the similarity measurement and the matching strategy of high-dimensional visual feature, a high-precision retrieval of brain image with semantics category was achieved, and the constructed disease category feature database of brain image was tested and evaluated through large numbers of pathological image retrieval experiments, the accuracy and the effectiveness of the proposed approach was verified.

**Conclusion:**

The disease category feature database of brain image constructed by the proposed approach achieved a quick and effective retrieval of images with similar pathological features, which is beneficial to the categorization and analysis of intractable brain diseases. This provides an effective application tool such as case-based image data management, evidence-based medicine and clinical decision support.

## Introduction

With the rapid development of medical imaging technology, various medical imaging devices are increasingly being used in clinical diagnosis, and about 80% of the data generated within a hospital are derived from the data of medical images. For example, in 2002, the Radiology Department of Hospital Medical Center of the Geneva University generated over 12,000 medical images each day in average. Thanks to the improvement in the performance of imaging equipment, the amount of data has increased exponentially [1]. These mammoth amounts of medical image data come from a variety of devices, such as ultrasound, X-ray computed tomography (CT), magnetic resonance imaging (MRI), digital subtraction angiography (DSA), and positron emission tomography (PET). In addition, they correspond to diverse kinds of human tissues and organs, such as the brain, chest, and lungs. Many kinds of diseases can occur to one organ. For example, brain diseases may include cerebral hemorrhage, pituitary tumor, and glioma etc. The enormous amount of data, a wide variety of imaging devices, various kinds of affected sites and different category of diseases create great challenges to the efficient organization and accurate retrieval of medical image data. Based on the existing PACS (Picture Archiving and Communication Systems) it was found that, constructing a more efficient and rational medical image feature database, organizing and managing these complex and diverse medical image resources for the purpose of classifying some intractable diseases are key measures to appreciate applications such as data management, evidence-based medicine, and case-based data management, evidence-based medicine and clinical decision support [2–4].

Convolutional Neural Network (CNN) is an effective feature extraction algorithm widely used in pattern recognition and image processing, and it is characterized by simple structure, few tuned parameters and strong adaptability. In recent years, a deep network using trained multiple stacked layers have drawn extensive attention [5]. Marked by ImageNet Challenge, deep learning algorithm has made great progress in the classification of large-scale color images. A variety of effective image classifiers have also been proposed successively, such as the classic AlexNet [6], GoogleNet [7] and ResNet [8]. The AlexNet architecture proposed by Krizhevsky et al. [6] in 2012 consists of 5 convolutional layers and 3 full convolutional layers. In the ImageNet large-scale visual recognition challenge, a set of data of 1.2 million images are divided into 1000 different categories, and the models that were designed have achieved substantial improvements in recognition performance compared to other scholars' models. In spite of a large amount of studies recently carried out on deep convolutional neural networks for the classification of color images, when these classic models are directly applied to medical images, the accuracy obtained is far from being compared to that of the color image classification of natural scenes. This is mainly due to the intrinsic particularity of medical images compared to natural color images. Due to the following inherent characteristics of medical images: heterogeneity, domination of grayscale images, high resolution, ambiguity, multimodality, etc., plus the immense visual differences of medical images and physiological and pathological information at different anatomical parts, the imaging knowledge relied on by diagnosis also varies. This attributes leads to a huge difference between them, and it also presents certain hindrances to the direct application of classic mature models such as AlexNet [9, 10].

Obtaining effective low-level visual features is the basis of achieving accurate classification and constructing a medical image feature database according to the category of disease, and is also the key to get the retrieval results quickly and accurately based on the excellent retrieval strategy [11]. In recent years, deep learning method has replaced the hand crafted feature extraction method which has been developed for past decades years, and become a new method of medical image visual feature extraction and representation [12–21]. Shin et al. [14] summarized three methods of learning medical image features by convolutional neural networks (CNN). The first is to train CNN model only with medical images, and adopt various techniques to avoid over fitting. For example, NovaSearch [15] used dropout, data augmentation and other technologies to process small sample sets in the ImageCLEFmed competition, and used medical image data to train CNN model. In reference [16], a classification system of Interstitial Lung Diseases (ILDs) based on convolutional neural network was proposed. Their dataset consists of seven classes, out of which six were ILD patterns and a health tissue class. They achieved 85.5% classification performance in characterizing lung patterns. Qayyum et al. [17] proposed propose a framework of deep learning for CBMIR system by using deep convolutional neural network (CNN) that is trained for classification of medical images. An intermodal dataset that contains twenty-four classes and five modalities is used to train the network. The learned features and the classification results are used to retrieve medical images. The second is to use the transfer learning to fine tune the CNN model trained in advance on the natural image using the medical image. For example, Bar et al. [18] used the transfer learning to learn the characteristics of chest image from CNN model trained in advance in natural image set. The third is to use the CNN model trained in advance to extract features, and then fuse with the traditional features to obtain more accurate retrieval and classification results [19].

Compared to the existing methods, our algorithm has the following characteristics: (1) Based on the deep convolutional neural network, an image classifier applicable to brain disease was designed to distinguish between the image features of different brain diseases with similar anatomical structures. Through the extraction and analysis of visual features of the image, each image was labelled with the semantic features categorizing a specific disease, which established the association between the visual features of brain image and the semantic features of the disease category, and a brain image disease category feature database can be constructed. (2) Based on the similarity measurement of high-dimensional visual feature and the matching strategy of category semantics, the high-precision retrieval of brain image with category semantics was achieved, and the constructed brain image feature disease category database was tested and evaluated through large numbers of pathological image retrieval experiments, the accuracy and effectiveness of the proposed approach was verified. The approach proposed in this paper will reveal an impending support for clinical decision and other applications based on the category of brain image feature diseases.

## Methods

### Ethics statement

All of the research methods and investigational tools in this study were approved by the Ethics Committee of Chinese Academy of Medical Sciences. All of the respondents in this manuscript gave a written informed consent to participate in the study, provided consent before filling out the questionnaire, and consented to the publication of the data. Ethical issues (Including plagiarism, informed consent, misconduct, data fabrication and/or falsification, double publication and/or submission, redundancy, etc.) have been completely observed by the authors.

### Extraction of brain image visual features based on deep convolutional neural network

Usually there are large differences between natural scene images. These differences are mostly represented by a global macroscopic feature. Therefore, when processing the natural scene images, all the networks designed by scholars usually layer-by-layer shrink the feature map, so that an overall feature can be gathered step by step, and this overall feature happens to be the most effective feature for distinguishing between different scenes. However, for medical images, especially for different disease grouping of the same organ, global feature is not necessarily the most effective feature. For example, for CT slices of the brain, their structures are similar from a macroscopic point of view, and the disease is represented just by a small block in the brain scan image [22].To distinguish between different disease groupings, the characteristics of these blocks remain as the important tools. If a classification network used in the natural scene image is employed, the information enclosed in these cell blocks may be gradually lost during the pooling process, reducing the accuracy of the final classification. In order to address this issue, the deep network designed in this paper can extract both the global and local features to achieve the classification by disease. The global features are conducive to distinguishing between slices at different directions or different angles, while the local features are useful for the correct judgments on the categories of different diseases in the same slice.

#### Basic module design

Based on the characteristics of medical images, the paper proposed a network for extracting features from images at multiple scales, which means it can be used to extract macroscopic global features and extract local pathological features. Finally, these global and local features are integrated through the fully connected layers to acquire the visual features that are eventually used for classification. The extracted features concurrently contain the contour information and the local texture information, with reasonably low information loss and a distinguishingly strong capacity of expression. The basic modules of the network used in this article are as follows:

This basic structure is a residual module and is an alternative of the legendary ResNet basic module. Compared to the basic module of ResNet, the basic module in this paper can extract multi-scale features. In the main branch of this module, there are three uninterrupted convolutions. Since each convolution is a mathematical operation based on the previous convolution, the receptive fields of the three convolutions are progressively enlarged, so that the global features can be extracted step by step, and the *Concat* layer can combine these local and global features. Additionally, the existence of the a *Add* layer also has the function of assimilating features at different scales, and can also accelerate the convergence speed of the network.

#### Architecture design of the overall convolutional neural network

**Fig 1:**
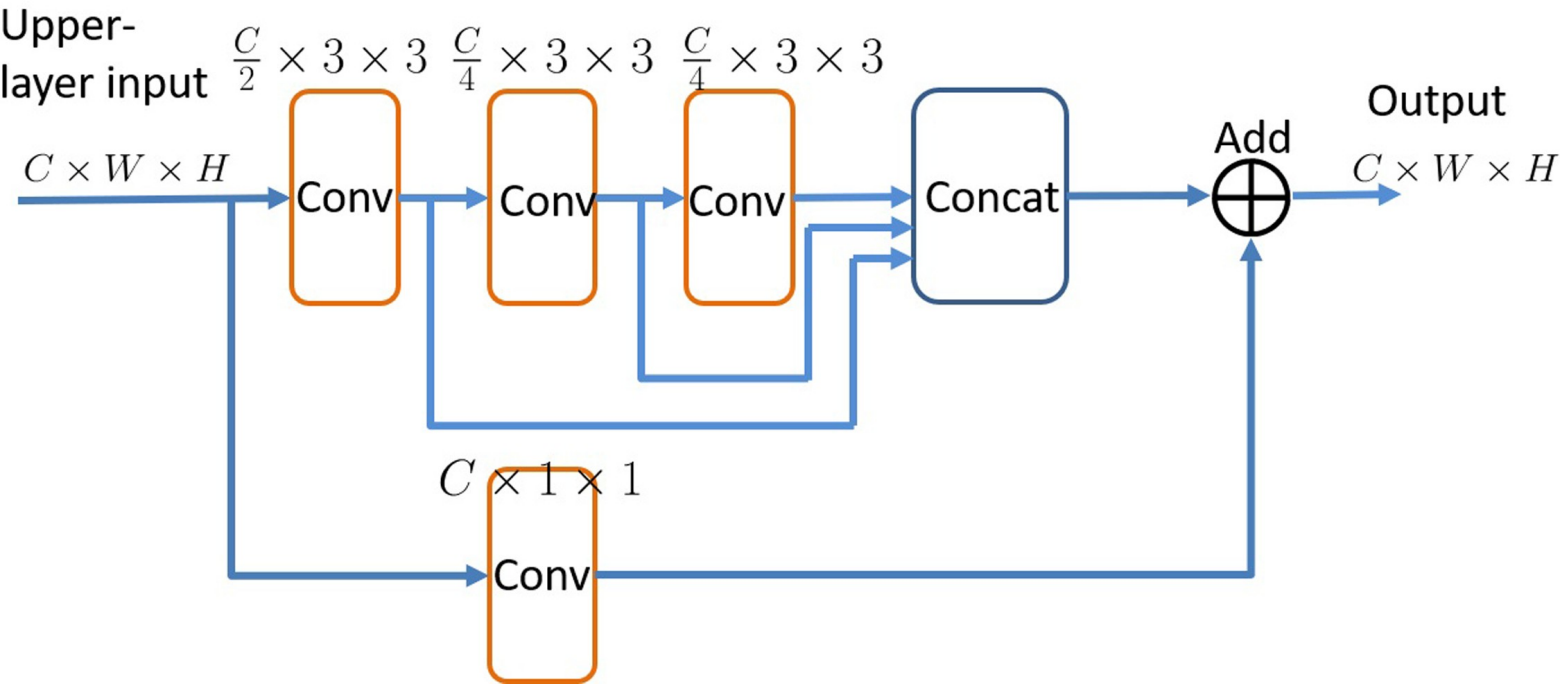
Basic module of the network.

**Fig 2:**
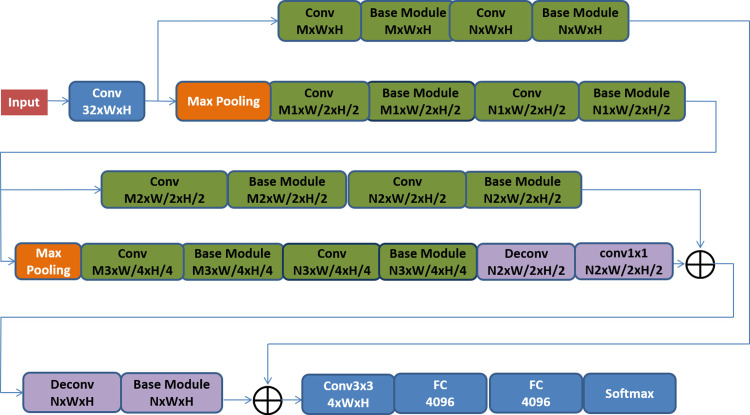
Complete network structure.

In addition to the multi-scale feature extraction in the basic module mentioned above, pooling layers was added in the network, and multiple basic modules were effectively combined with the pooled layers, which can further achieve more effective extraction of scale features. The complete network structure is shown below:

The Basic Module shown in Fig 2 stands for the basic block in Fig 1 *Mx* or *Nx* refers to the number of channels of the feature map and W and H refer to the value of width and height of the feature map at this layer. All convolutional layers use the3x3 kernel unless otherwise specified. The network used in this paper can be used to extract and merge the features of the image at the original scale, the 1/2 scale and the 1/4 scale, and it efficiently make use of the global and local features, so that the distinguishing feature and expression capacity can be made stronger.

#### Selection of classification function

Since the Logistic function can only be used in the binary classification problem, its polynomial regression, named the *Softmax* function, can solve the multiclass classification problem. The Logistic function of course, can also be used to create ***k*** independent binary classifiers to address multiclass classification problems. However, because the classification of brain image disease categories is a mutually exclusive relationship. *Softmax* function is more suitable to serve as a classification function in this case.

The *Softmax* function is actually a normalized exponential function, which is defined as follows:

$$P(i)=\frac{\exp(\theta_{i}^{T}x)}{\sum_{k=1}^{K}\exp(\theta_{k}^{T}x)}$$ (1)

where ***θ***_***i***_ and ***x*** are column vectors, and ***θ***^***T***^_***i***_***x*** may be replaced by a function ***f***_***i***_***(x)*** with respect to ***x***. With the *Softmax* function, the variety of ***P(i)*** can be made between [0, 1]. In the regression and classification problems, ***θ*** is the target parameter; by seeking, calculation is carried out to make the maximal ***θ***_***i***_ of ***P(i)*** as the optimal parameter.

### Construction of the brain image diseases category feature database

Using the deep network neural structure designed in this article, the characteristics of the category features of disease enclosed in brain images were extracted, and the mapping model of the visual features to the semantic features of disease categories was constructed. This establishes the relationship between the visual features, the semantic disease category, and the construction the category of the database of brain image feature disease, providing assurance to the efficient retrieval.

**Fig 3:**
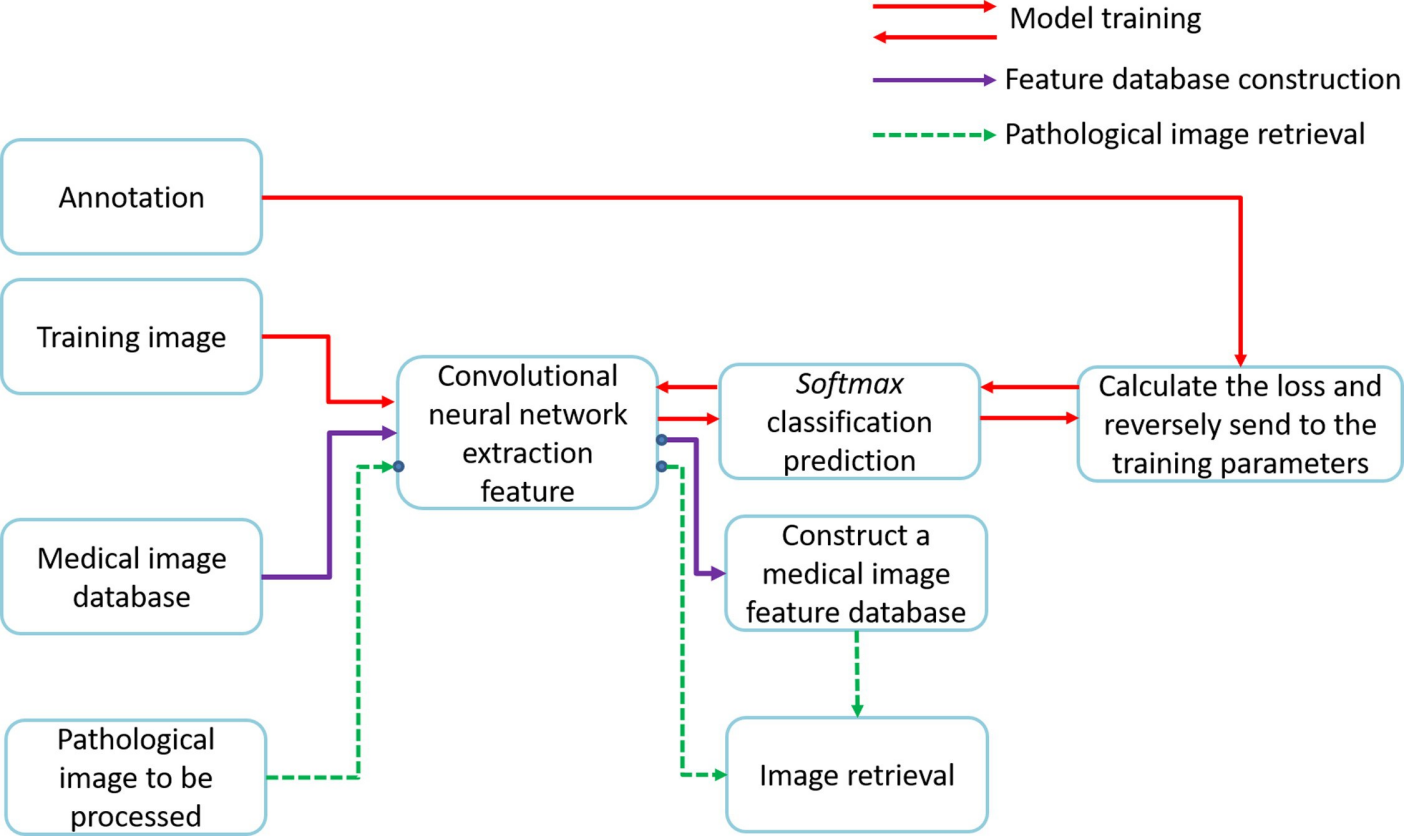
Procedures of constructing the brain image feature database and retrieving the pathological images.

The flow chart of constructing the brain image feature database and retrieving the pathological images adopted in this article are as follows:

Fig 3 shows the main procedures of constructing the brain image feature database and retrieving the pathological images designed in this article, and it consists of three parts:

(1) The procedures indicated by the red arrow represents the training process, and its input is a batch of annotated training images. The deep convolutional neural network extract the features of the training images under the current parameters and then the features are feed into the *Softmax* classifier for test. The predicted outcome are compared with the annotated labels to calculate the differences in classification, and then the network parameters are adjusted through the back propagation algorithm. After several rounds of iterations, the well-trained parameters can be obtained. The reason why a classification task is adopted to train the network is that there are to some extent similarities between the semantic features extracted by the classification and retrieval approaches.

(2) The procedures indicated by purple arrows stands for the feature database construction process, and its input is a scanned brain image in the medical image database. These images can be feed into the well-trained network to calculate the corresponding features. These features obtained by the original network can be used directly, or can be processed by other algorithms as needed, such as Hash transformation. By storing these features into the database, a brain image feature database is constructed.

(3) The procedures indicated by the green arrow represents the image retrieval process. The features of the pathological images to be processed can be obtained through network calculation, and then compared with the features in the feature database, so that the pathological image is retrieved as a reference for auxiliary diagnosis.

## Experiments and result analysis

### Experimental data and experimental environment

The images of three brain diseases were collected to serve as the training data. The first data corresponded to the images of 200 cases of cerebral hemorrhage and pituitary tumors collected from the Imaging Department of a third-grade hospital, which include 225465 and 235757 image data, respectively. The image modes included CT and MR. Another image data of CT and MR models of 199 patients (41183 images) were collected from the open Cancer Imaging Archive (TCIA) created by the National Cancer Institute (NCI). These training data were used to construct a disease category feature database corresponding to the brain image.

The experimental platform used in this study was the Intel (R) Core (TM) i3 CPU (2.53GHz), 4G memory, and Python language is used to conduct the experiment.

### High-dimensional visual feature similarity measure

The original feature generated by the network is vector of 4096 dimensions. Due to the high dimension, the calculation is overloaded during the search, especially when the size of the database is large. A large amount of calculations will obviously affect the retrieval efficiency and affect the user experience. However, in our application, the 4096-dimensional vector has some redundancy in information representation, so the dimension of the vector can be further reduced. In this paper, the Principal Component Analysis (PCA) method is used to reduce the dimension of the vectors. As a method used to analyze data in multivariate statistical analysis, Principal Component Analysis (PCA) describes the sample with a small number of features for the purpose of reducing the dimensionality of the feature space. The principle involved is to make a high dimension vector **x** projected into a low-dimensional vector space through a special eigenvector matrix **U**, which is characterized as a low-dimensional vector **y**, with only part of secondary information being lost. In other words, the vector characterized by low-dimension and the eigenvector matrix can basically reconstruct the corresponding original high-dimensional vector. This can be achieved by embracing the optimal orthogonal transformation algorithm. Through the SVD decomposition of the covariance matrix, we can obtain the eigenvalue, eigenvector matrix, and the high-dimensional vector can be transformed into the low-dimensional vector by the eigenvector matrix. After the dimension-reduction processing, the 1024-dimension vector feature was finally adopted to describe the visual features of the image.

During the searching process, a calculation of similarity is performed on the 1024-dimensional features and the feature subset to be matched in the feature database through the Euclidean distance of the vector.

$$S_{v,v_{0}}={\left\|v-v_{0}\right\|}_{2}$$ (2)

where *v*_0_ is the calculated eigenvector, *v* is one of the eigenvectors in the feature database. The correspondence distances are then sorted in ascending order, and a list of retrieved image is obtained corresponding to the reference image. The image with the shortest distance represents the image with the highest similarity, which will be output as the retrieved image.

### Evaluation method

Precision and recall are currently widely used evaluation criteria for measuring the effects of image retrieval. The formula is as follows:

P=a/(a+b),R=a/(a+c) (3)

where ***P*** represents the precision, ***R*** represents the recall, ***a*** represents the number of images that satisfy the condition of retrieval, and ***b*** represents the number of images that fail to satisfy the condition of retrieval, while ***c*** represents the number of images in the system that satisfy the condition but are not retrieved.

A higher accuracy and retrieval rate means an better retrieval result of the system. In general, the retrieval rate and accuracy are contradictory. When the accuracy is required to be relatively high, the retrieval rate will be low, and vice versa. Therefore, when the general retrieval system only requires an optimal balance between the two, it means that a relatively good retrieval performance is achieved. The recall-precision rate is applied to reflect the overall performance of the entire retrieved results. But it is not sensitive to the different ranks in the retrieval results. To tackle this issue, a set of the Mean Average Precision (MAP) in a group of inquiry cases and the mean recall rate were adopted as measures. On one hand, it can improve the sensitivity of ranks, and on the other hand, it can reduce the instability of the evaluation caused by inquiry cases. The MAP calculation formula is as follows:

$$\begin{array}{l} AP(q)=\frac{1}{N_{R}}\sum_{n=1}^{N_{R}}P_{q}(R_{n})\\ MAP=\frac{1}{\left|Q\right|}\sum_{q\in Q}AP(q) \end{array}$$ (4)

where *P_q_*(*R_n_*) represents the value of the precision *P_q_* when the corresponding recall is *R_n_*; *Q* represents the inquiry case set; |*Q*| represents the number of inquiry cases.

### Analysis of results

**Fig 4:**
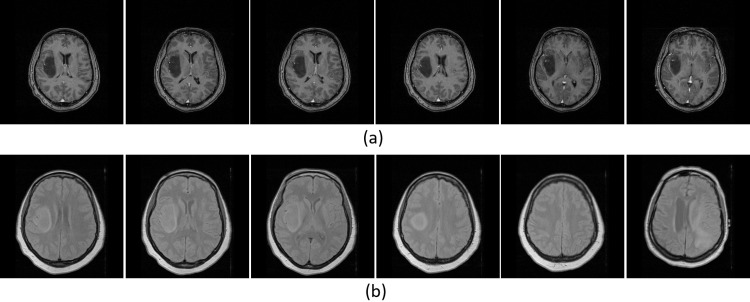
Example of two sets of retrieval results.

From the images of the three brain diseases collected in this paper, 200 images of each disease image were selected as the testing data. Fig 4 shows two results obtained by the image retrieval method proposed in this paper. Among of them, the leftmost image is the retrieval image, and the remaining five images on the right are the results retrieved from the image database. The retrieved results are very similar to the given retrieval images. It indicates that the proposed network is very effective in medical feature extraction.

Table 1 shows the average recall and precision of 200 cases selected through the retrieval from each of the three diseases as the inquiry cases.

**Table 1:** Mean precision and recall.

Focus-containing brain image	Mean precision	Mean recall
Brain hemorrhage image	80.4%	82.6%
Hypophysoma image	89.3%	85.5%
Glioma image	85.8%	80.9%

We compare the results of medical image retrieval based on deep convolution neural network shown in reference [17], as shown in Table 2. Of course, as mentioned by the author of reference [17], there is no available standard medical data set that can be used to benchmark the retrieval system, so it cannot be directly compared. MAP is selected as the comparison standard of retrieval precision, and a higher MAP value is obtained. Although the images we retrieve only include CT and MR modalities, while the images retrieved in reference [17] include MR, CT, PT, PET, and OPT modalities, our algorithm processes a large number of images, and only involves the retrieval of brain images with relatively high similarity among three different diseases.

**Table 2:** Comparison of methods.

Method	Images	Modalities	Classes	MAP
Our method	702405	MR, CT	3	0.85
Ref. [17]	7200	MR, CT, PT, PET, OPT	24	0.69
Ref. [23]	14410	X-Ray	57	0.86

### Retrieval interface

**Fig 5:**
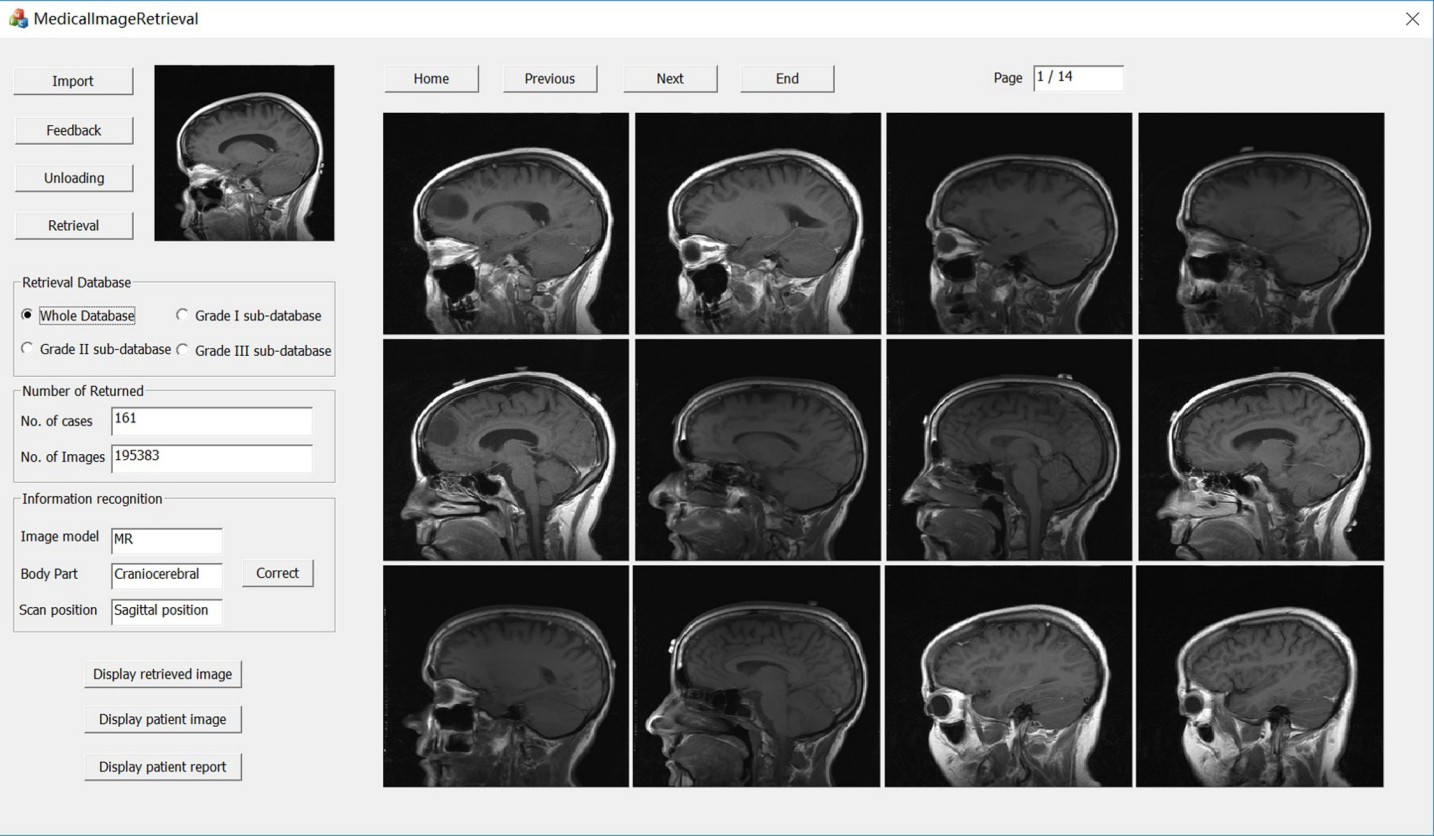
Results of retrieval results of inquiry case map.

Based on the extracted brain image visual features and the constructed brain image category feature database, the retrieval of brain images containing the information and characteristics of similar disease category in the inquiry example map (undiagnosed) was implemented. The retrieval interface is shown in Fig 5 The right side of the figure shows the representative images of 161 cases of the same disease type retrieved from the inquiry case map of a glioma patient. Each case only shows the best matching image. The total number of corresponding images to these 161 cases is 195383. Other images can be checked by selecting a certain case, and clicking the button “Display patient image”, or click the button “Display patient report” to view the corresponding patient’s examination report. The 161 cases shown on the right are sorted according to the degree of similarities with the outputs displayed; for the most facade cases, namely the cases with a relatively high similarity, can be used to assist the clinician's diagnosis.

## Conclusion

In clinical practice, doctors not only aspire to retrieve images of brain tumors with overall similarity from the image database, but also yearn to retrieve images showing the same location of lesion and the nature of consistent tumors, which tends to put a higher requirement for image detection tasks. In this regard, the paper proposed a method for constructing a disease category feature database of brain image based on convolutional neural network. The convolutional neural network can be used to extract the visual features corresponding to medical images on multi-scale images. Based on the constructed visual features database, the brain images with similar case characteristics in inquiry case maps can be quickly and accurately retrieved, providing reference to assist clinicians' diagnosis. The future study will be focused on combining the above knowledge with the knowledge in clinical field, taking into consideration the spatial location, the nature of the tumor, thereby further improving the mean precision and recall rate of retrieval images, so as to better satisfy the needs of clinicians.
